# Supplementary material for: Conditional chemoconnectomics (cCCTomics) as a strategy for efficient and conditional targeting of chemical transmission
Source: eLife. 2024 Apr 30;12:RP91927. doi: 10.7554/eLife.91927 (PMC11060718; doi:10.7554/eLife.91927)
Supplement: Figure 3—source data 3. [file elife-91927-fig3-data3.zip › Figure 3 source data3 part 1.zip!/Figure 3-Source data 3-part1/1175-9-CRISPResso_Report_rtnd62ko.zip!/CRISPResso_on_1175_10/3a.Indel_size_distribution.pdf]

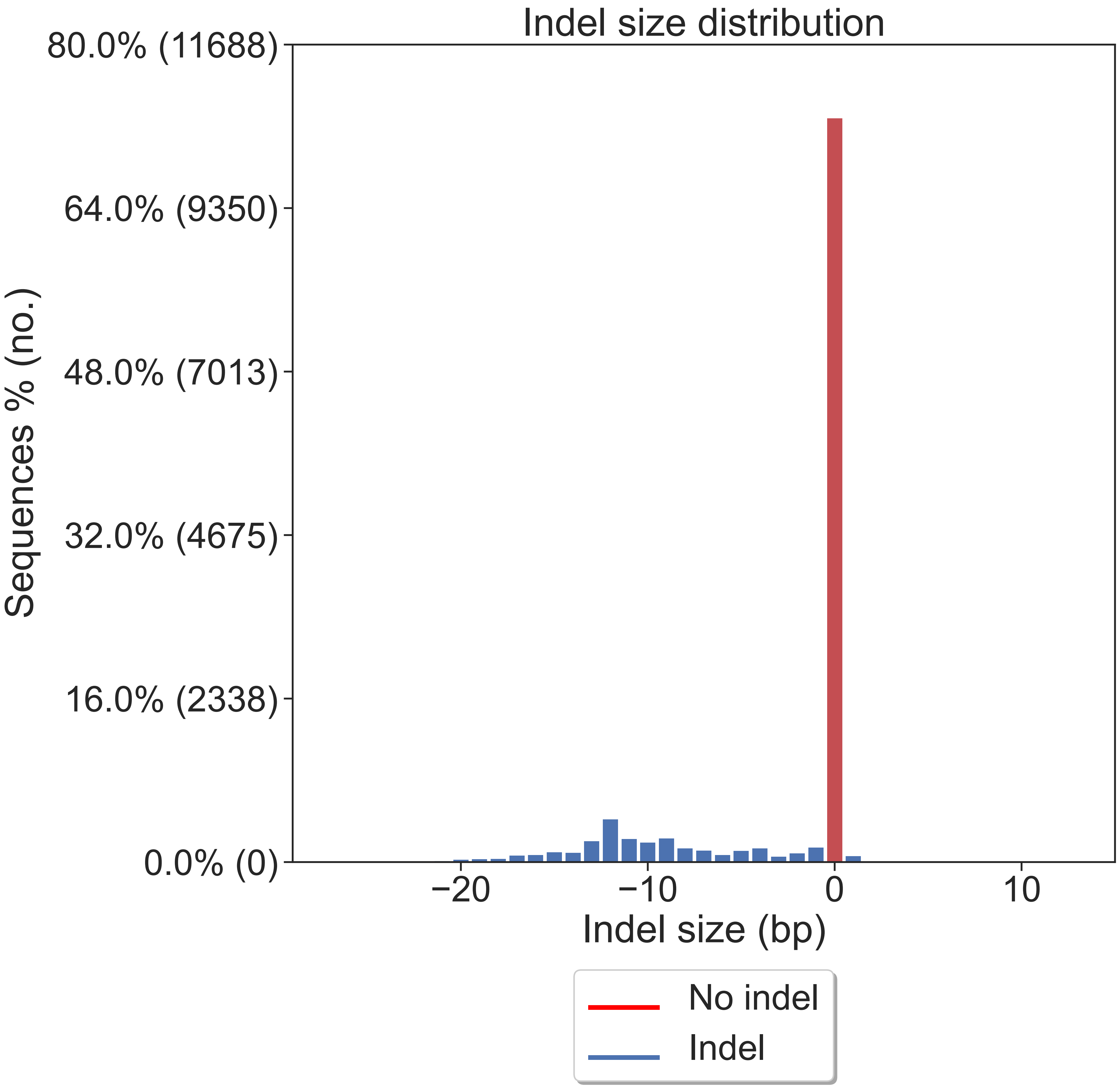

Indel size distribution
80.0% (11688)
64.0% (9350)
48.0% (7013)
32.0% (4675)
16.0% (2338)
0.0% (0)
Sequences % (no.)
−20
−10
0
10
Indel size (bp)
No indel
Indel
